# Post-Impact Fatigue Damage Monitoring Using Fiber Bragg Grating Sensors

**DOI:** 10.3390/s140304144

**Published:** 2014-03-03

**Authors:** Chow-Shing Shin, Shien-Kuei Liaw, Shi-Wei Yang

**Affiliations:** 1 Department of Mechanical Engineering, National Taiwan University, Taipei 10617, Taiwan; E-Mails: skliaw@mail.ntust.edu.tw (S.-K.L.); R96522520@ntu.edu.tw (S.-W.Y.); 2 Department of Electronic Engineering, National Taiwan University of Science and Technology, Taipei 10607, Taiwan

**Keywords:** fatigue, impact damage, polymer-matrix composites, health monitoring, fiber Bragg grating

## Abstract

It has been shown that impact damage to composite materials can be revealed by embedded Fiber Bragg Gratings (FBG) as a broadening and splitting of the latter's characteristic narrow peak reflected spectrum. The current work further subjected the impact damaged composite to cyclic loading and found that the FBG spectrum gradually submerged into a rise of background intensity as internal damages progressed. By skipping the impact, directing the impact to positions away from the FBG and examining the extracted fibers, we concluded that the above change is not a result of deterioration/damage of the sensor. It is caused solely by the damages initiated in the composite by the impact and aggravated by fatigue loading. Evolution of the grating spectrum may therefore be used to monitor qualitatively the development of the incurred damages.

## 1. Introduction

Composite structures such as aerospace vehicles and wind turbine blades are susceptible to impact damage caused by careless handling during manufacturing and maintenance and by foreign object impacts such as bird-strikes and hailstorms during service. Such damages involve a number of different failure mechanisms such as delamination, debonding, fiber breakage and matrix cracking [1]. The damages caused are often insidious without leaving marked evidence on the impact-suffering surface [2]. Although small in extent, these microscopic damages can lead to deterioration of mechanical properties [3–5]. In particular, on subsequent cyclic service loading, these microstructural defects may grow and eventually lead to catastrophic failures. Current non-destructive examination techniques are only sensitive to some of these failure mechanisms and are responsive only when the defects reach a certain size. It is often impracticable to use these techniques for close monitoring of the development of these defects. Recently, there are general interests in the development of integrating fiber optic sensors into composite structures for structural integrity monitoring. Optical fiber has a small diameter, long fatigue life and may be embedded inside a composite material and literally come into contact or at least into very close proximity of the internal defects. Fiber Bragg Grating (FBG) is one of such sensor elements that has been shown to be able to detect impact damages in composites [5–11] and monitor impact event occurrence [12–14].

The output from an FBG is the wavelength shifting and shape changing of its characteristic reflected spectrum. There is no simple correlation between the spectrum changes and the damage mechanism involved. The techniques employed for FBG sensor to detect impact damages fall into three categories. The first technique makes use of residual strain changes [5–7]. Residual stress/strain invariably exists inside fiber reinforced polymeric composites due to differential thermal contraction after curing. This residual strain field will be perturbed by the microscopic damages brought about by an impact. Residual strain changes are reflected as a shift in the peak wavelength of the FBG spectrum. The second technique monitors the appearance and growth of delamination [8]. In this method, the FBG sensors must be deployed on the delamination interface. As impact caused a delamination to extend across part of the FBG, strain on the debonded section of the sensor is largely relieved while the bonded portion is still under strain. This is reflected as a shifting and chirping of the FBG spectrum. A third technique used the FBG as an ultrasonic wave receiver. Ultrasonic waves are usually generated by a piezo-actuator. On encountering a delamination, the properties of the wave changes and is picked up by the FBG [9–11]. FBG has also been shown to be able to monitor the progressive damage due to repeated impacts [12] and compression after impact [5]. Similar study on the monitoring of damage development during post-impact fatigue is still lacking. The latter is deemed important as an impact-damaged composite structure often needs to face cyclic service loading in practical applications. In the current work, we investigated the feasibility of employing FBG sensor to monitor defect growth during post-impact fatigue loading.

## 2. Material and Methods

When a broadband light is coupled into an optical fiber with a uniform Bragg grating, a single peak with wavelength λ satisfying the Bragg diffraction criterion will be reflected:

(1) $$\lambda =2{\text{n}}_{\text{e}}\Lambda$$

where *n_e_* is the effective refractive index and Λ is the periodicity of the grating. When either or both of the *n_e_* and Λ change, the center wavelength of the reflected spectrum shifts. Λ will be changed if the FBG is subjected to a deformation. Such deformation may be caused by mechanical or thermal strains. *n_e_* will be affected by variation in temperature and the triaxial stress state acting on the fiber. If the uniformity of the grating period is perturbed, the single peak reflected spectrum will broaden or chirped. In general, the reflected wavelength will shift by ∼1 *pm* under a strain of 1 με. or a temperature change of 0.1 °C. If temperature variation is negligible, then the change in the spectrum basically reflects a change in the stress/strain status along the FBG.

**Figure 1.**
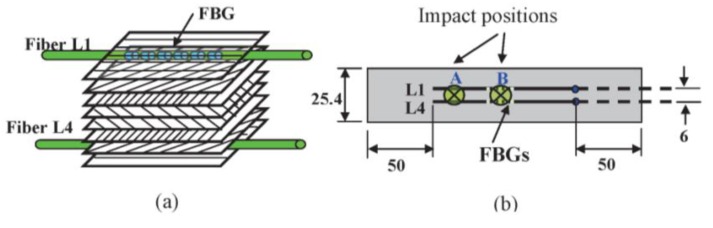
(**a**) Schematic lay out of the prepreg stacking and the embedded optical fiber sensors; (**b**) positions of impact relative to the fiber sensors.

Quasi-isotropic laminates with T300/3501 Graphite/Epoxy prepreg stacked in the sequence [0/45/90/−45]_s_ were cut into specimen coupons (200 × 25.4 × 1 mm). FBG sensors were embedded right under the two outer 0° laminae along the axial loading direction as shown in Figure 1a. Each of the fibers was offset by 3 mm from the centerline of the specimen. The fibers were led into the specimen coupon at one side and terminate inside the specimen at some distance short of the gripping position at the other side, as shown in Figure 1b. FBGs were fabricated in a Ge–B co-doped single mode fiber by side writing using a phase mask with a period of 1.05 μm. The sensing length of the FBGs was about 10 mm. The reflectivity of the resulting FBG was about 99%, and the peak wavelengths were between 1,550 and 1,551 nm. The full width half maximum (FWHM) of the FBGs was about 0.175 nm. Impacts were made at either of the two locations designated A and B in Figure 1b, using a 260 g aluminum weight falling from a height of 140 cm with an apparatus that conforms to ASTM D5628. B was the position of the FBG and position A was 30 mm from B. The fiber on the side that faced the impact was designated L1 and the one on the back surface L4. After impact the coupons were subjected to cyclic fatigue loading from 0.5 to 5 kN at a frequency of 5 Hz using an MTS servo-hydraulic testing system 810 (MTS Systems Corporation, Eden Prairie, MN, USA) for 200,000 cycles. The reflected spectra from the FBGs were interrogated periodically using an optical spectrum analyzer (Anritsu MS9710C OSA, Anritsu Company Ltd., Kanagawa, Japan) under the load-free condition. The above tests have also been repeated on specimens without undergoing any impact to serve as control. Ultrasonic C-scan was employed to examine the impact damage before and after the fatigue test. After testing, destructive sectioning of the tested specimens were made to allow internal damages to be observed under an optical microscope. Fiber extraction was attempted in order to examine the integrity of the FBGs after the impact and fatigue loading.

## 3. Results and Discussion

### 3.1. Fatigue without Impact

**Figure 2.**
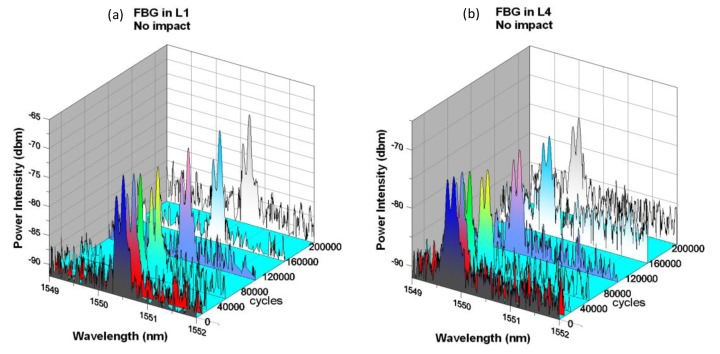
Spectra development from embedded FBGs in (**a**) L1 and (**b**) L4 with fatigue cycles in specimen without impact.

Figure 2 shows the FBG spectra from L1 and L4 fibers at different loading cycles in a composite specimen subjected to cyclic loading without suffering an impact. Originally each of the FBG spectra has a single sharp peak. On embedding, curing and cutting into testing coupons, a shift in peak wavelength together with slight widening and splitting of the peak occurred due to residual stresses induced during the above fabrication processes. In the course of 200,000 cycles of fatigue loading, the FBG peaks drifted gradually and slightly to longer wavelengths but retained their shapes and intensities throughout. This suggests that the current level of fatigue loading alone did not cause significant damage in the composite material and so the status of the FBGs in the specimen was not perturbed.

### 3.2. Fatigue Following Impact at Position B

**Figure 3.**
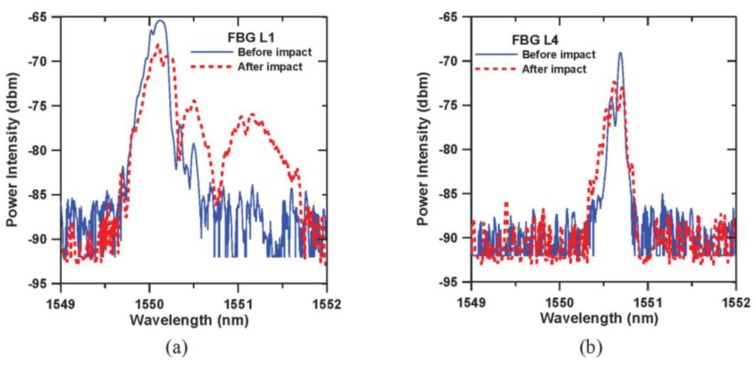
(**a**) L1 and (**b**) L4 FBG spectra before and after impact damage.

Figure 3 shows the FBG spectra for the fibers L1 and L4 just before and after impact. The solid lines in Figure 3 are the FBG spectra following composite testing coupon preparation. After applying the impact, the original peaks widened and split heavily into a number of distinct peaks (broken lines in Figure 3). The above phenomena are more prominent in the spectrum from the L1 fiber, which is closer to the impact face. Such a change in the spectra suggests the strain distribution along the FBG has changed from roughly uniform to non-uniform. The latter is probably caused by the impact induced internal defects that have perturbed the residual stress field near the FBG.

**Figure 4.**
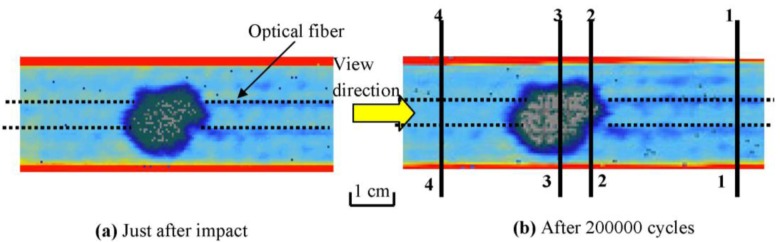
Ultrasonic C-scan images of post impact damage at (**a**) just after impact and (**b**) after 200,000 cycles.

**Figure 5.**
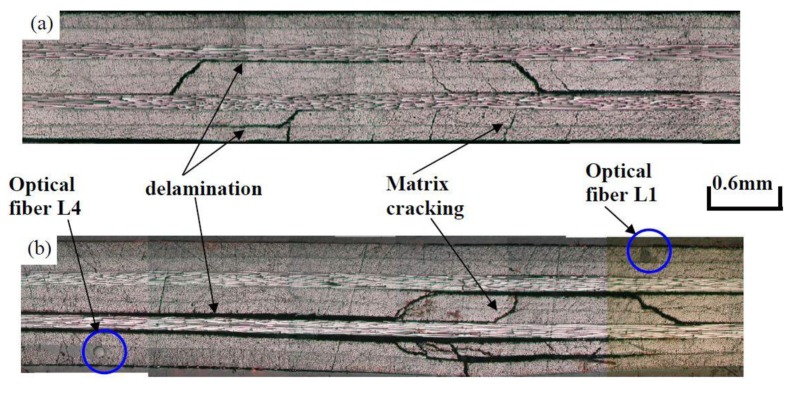
Optical micrograph of the Section 2-2 showing the positions of the fiber sensor and the extent of post-impact damage (**a**) just after impact; (**b**) after 200,000 cycles.

The impact damaged specimen was then subjected to cyclic loading. Figure 4 compares the internal impact damage as visualized by ultrasonic C-scan before fatigue testing and after 200,000 loading cycles. C-scan reflected the occurrence of delaminated interface perpendicular to the ultrasonic beam inside a composite material. Slight enlargement of the damaged area can be seen on comparison of Figures 4a,b. The extents of damage at a number of Sections (1.1 through 4.4 in Figure 3) were examined in a direction along the fibers in Figure 4 under an optical microscope. Figure 5 shows typical comparison of observed damages before and after 200,000 fatigue cycles at Section 2.2. Note that the specimen in Figure 5a is different from that in Figure 5b as this examination is destructive. Delamination and cracking in the matrix occurred extensively in the lower half of the section, which faced tensile stresses during the impact. Marked aggravation of these damages during the cyclic loading was evident (Figure 5b). In Sections 1.1 and 4.4, delamination was not seen. Matrix cracking was not observed initially but very sparing occurrence could be spotted after 200,000 loading cycles. Comparison of Figures 4 and 5 shows that although ultrasonic C-scan can reveal the extent of delamination damage, it is not a sensitive tool to monitor the growth in severity of such defects. Also it cannot detect the occurrence of matrix cracking.

**Figure 6.**
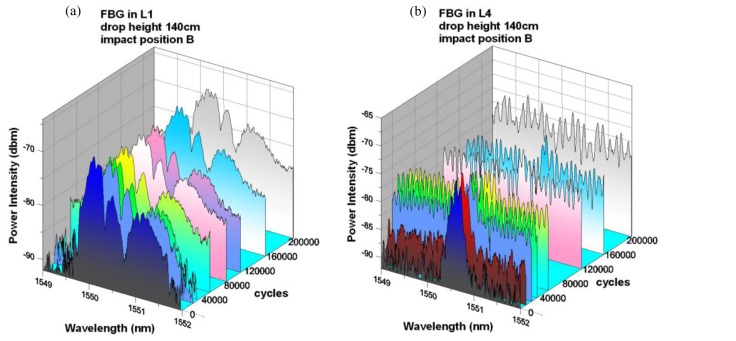
Spectra changes from embedded FBGs in (**a**) L1 and (**b**) L4 with various fatigue cycles under a 140 cm drop height and impact at position B.

The FBG spectra at various loading cycles are shown in Figure 6. On cycling, the peaks in the L1 spectrum continued to widen gradually. At 50,000 cycles, a rise in the intensity of the background wavelengths became obvious. At the end of 200,000 cycles, the original peaks can still be recognized though they are not as distinct as before due to the rise in intensity of the background wavelengths. For the L4 spectrum, the intensity of the wavelengths surrounding the original peak has already risen by a significant amount at 5,000 cycles. It continued to rise rapidly with the increase in fatigue loading cycles. At the end of 200,000 cycles, the original reflection peak was no longer discernible in the spectrum. A possible cause of the gradual widening and submersion of the spectra peaks was the continuous development and spread of the original impact damage in the composite. Such damage perturbed the strain field and caused steep strain gradient in the vicinity of the FBGs. In this case, the evolution of the FBG spectra may be employed to monitor the aggravation of the impact damage in a qualitative manner. Quantitative correlation is deemed impracticable as the damages involved are complicated and may vary from case to case.

It should be pointed out that that the observed changed in the spectra could also be caused at least partly by the development of damage in the optical fiber. The impact and subsequent cyclic loading might induce defects and microcracks in the optical fiber. Such microcracks could cause stress concentration which alters the localized refractive index through the photoelastic effect [15] and perturbs the periodicity of the grating. Moreover the flat smooth end of a nicely fractured fiber may act like a mirror which reflects light without discrimination on the wavelength. Both effects could lead to the rise in background intensity and the disappearance of the FBG reflection peak. To clarify this, we tried to extract the fibers from the tested specimens to examine their integrity.

### 3.3. Extracting and Examining the FBG from the Post-Impact Fatigued Specimens

**Figure 7.**
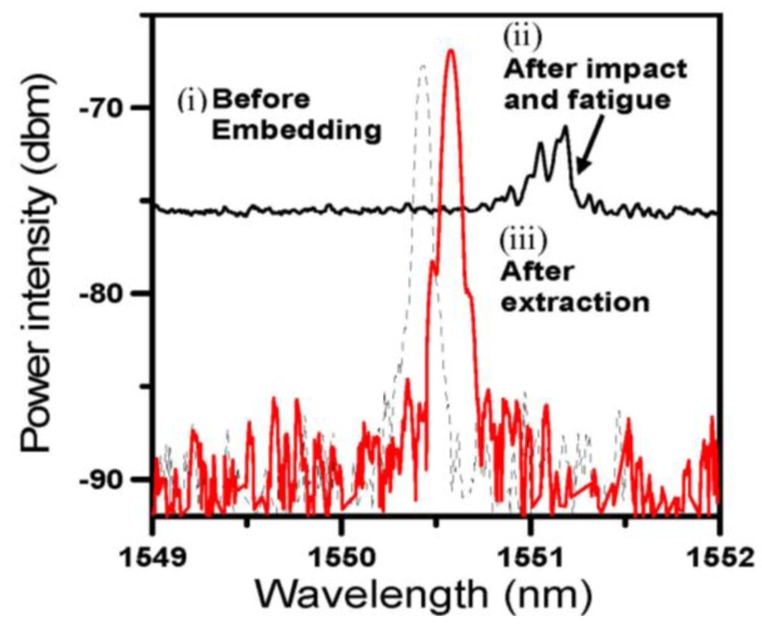
Comparison of FBG spectra (**i**) before embedded in the composite specimen; (**ii**) after post impact fatigue for 200,000 cycles and (**iii**) after fiber extraction from the post impact fatigued composite specimen.

By soaking the post-impact fatigued specimens in acetone for 1 day, the resin was softened to a great extent. During this process, it was found that the characteristic Bragg spectrum has partially been restored. By carefully dissecting the softened specimens to draw out the optical fibers, we found that the fiber appeared intact under an optical microscope. Moreover its characteristic FBG spectrum has basically been fully restored (Figure 7). Both the L1 and L4 FBGs behaved similarly and only the L4 spectra are shown here.

**Figure 8.**
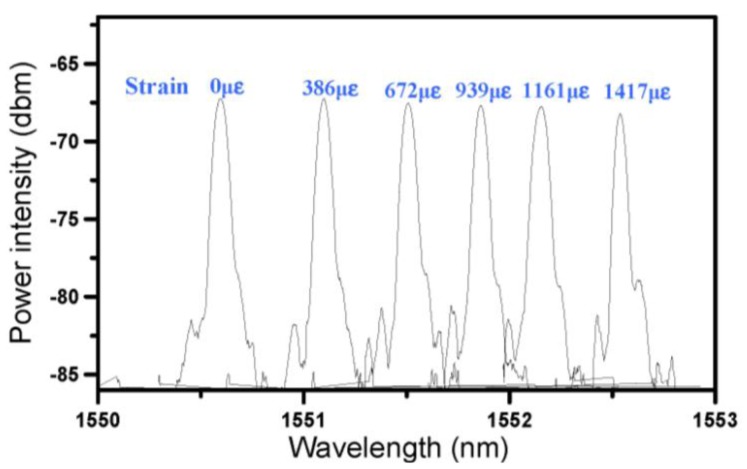
Shifting of FBG spectrum when the optical fiber was subjected different strains on a spring steel strip.

To thoroughly check whether there was any defect or microcrack induced in the optical fiber, the extracted fiber was stuck using cyanoacrylate adhesive on a thin spring steel strip. The later was then bent to different curvatures while the FBG spectra were monitored. A strain gage stuck alongside the FBG recorded the strain at each curvature. Figure 8 shows the L4 FBG spectra at different applied strains. It can be seen that the Bragg reflection peak shift to the right in proportion to the applied tensile strain. The shape of the peak maintained the same without any distortion, chirping or rising of the background intensity. This proves beyond doubt that the FBG sensor is defect-free and that the submersion of the reflection peaks in Figure 6 can only be attributed to the steep strain gradient in the composite specimen in the vicinity of the FBGs. Such steep strain gradient has to be caused by the growing damages initiated by the impact and aggravated by the cyclic loading.

### 3.4. Fatigue Following Impact at Position A

**Figure 9.**
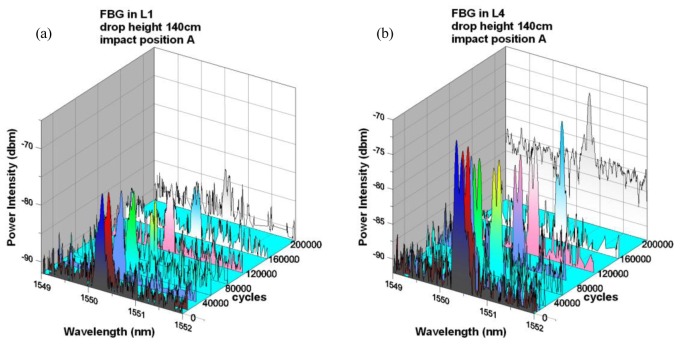
Spectra from embedded FBGs in (**a**) L1 and (**b**) L4, with fatigue cycles for impact at position A.

When impact was made at position A 30 mm from the FBG locations instead of B, the resulting spectra (Figure 9) evolved very slowly. Before 120,000 cycles, other than a gradual slight drop in intensities, the overall shape of the reflected peaks remained similar in both L1 and L4. More distinct changes in the peaks were observed at 160,000 cycles. At 200,000 cycles, a uniform rise in background intensity occurred in the spectrum from L4 (Figure 9b) but the original peak was still clearly discernible. This fading of the FBG peak may be explained by the development of the initial impact damage to such an extent that it was approaching the FBG and has exerted a certain degree of steep strain gradients on the matrix surrounding the FBG. When compared with Figure 6b, the background intensity rise was much delayed and less serious. This late and mild occurrence of background intensity rise corroborated with the previous microstructural observation that only sparing matrix cracking has been spotted 30mm away from the original impact site after 200,000 fatigue cycles.

Thus, evolution of the grating spectrum reflects qualitatively the development in severity and proximity of the incurred damages. It is much more sensitive than conventional non-destructive testing techniques such as the ultrasonic C-scan. With suitably deployed FBGs (say near the compressive face of an impact), one may be able to monitor status of structural health and give an alarm when the damage has grown beyond a certain size. The current tool will be especially helpful if one wants to monitor the effectiveness and long-term durability of a composite patch repair of the impact damage. More works are undertaken in these directions.

## 4. Conclusions

Embedded fiber Bragg gratings have been employed to monitor the post-impact fatigue damage development. It was found that the fiber Bragg grating sensor can survive a low velocity impact and the subsequent fatigue loading. Moreover, the evolution of the grating spectrum, which consists of a gradual submerging and eventual disappearance of the characteristic peak against a rise of background intensity, is caused by the growth of the internal damages and thus can be used to monitor qualitatively the development in severity and proximity of the damages incurred in the composite material during post impact service loading.

## References

[1] Talreja. R.R. (1987). Fatigue of Composite Materials.

[2] Cantwell W.J., Morton J. (1985). Detection of impact damage in CFRP laminates. Compos. Struct..

[3] Pintado P., Vogler T., Morton J. (1991). Impact damage development in thick composite laminates. Compos. Eng..

[4] Kim C., Jun E. (1992). Measurement of impact delamination by deply technique. Exp. Tech..

[5] Takeda S., Aoki Y., Nagao Y. (2012). Damage monitoring of CFRP stiffened panels under compressive load using FBG sensors. Compos. Struct..

[6] Chambers A.R., Mowlem M.C., Dokos L. (2007). Evaluating impact damage in CFRP using fibre optic sensors. Compos. Sci. Technol..

[7] Pearson J., Prabhugoud M., Zikry M., Peters K. *In-Situ* Failure Identification in Woven Composites throughout Impact Using Fiber Bragg Grating Sensors.

[8] Takeda S., Minakuchib S., Okabec Y., Takeda N. (2005). Delamination monitoring of laminated composites subjected to low-velocity impact using small-diameter FBG sensors. Compos. Part A.

[9] Tsuda H., Toyama N., Urabe K., Takatsubo J. (2004). Impact damage detection in CFRP using fiber Bragg gratings. Smart Mater. Struct..

[10] Takeda N., Okabe Y., Kuwahara J., Kojima S., Ogisu T. (2005). Development of smart composite structures with small-diameter fiber Bragg grating sensors for damage detection: Quantitative evaluation of delamination length in CFRP laminates using Lamb wave sensing. Compos. Sci. Technol..

[11] Lee J.R., Tsuda H., Toyama N. (2007). Impact wave and damage detections using a strain-free fiber Bragg grating ultrasonic receiver. NDT & E Int..

[12] Kuang K.S.C., Kenny R., Whelan M.P., Cantwell W.J., Chalker P.R. (2001). Residual strain measurement and impact response of optical fibre Bragg grating sensors in fibre metal laminates. Smart Mater. Struct..

[13] Chen B.L., Shin C.S. (2010). Fiber Bragg Gratings array for structural health monitoring. Mater. Manuf. Process.

[14] Shin C.S., Chen B.L., Cheng J.R., Liaw S.K. (2010). Impact response of a wind turbine blade measured by distributed FBG sensors. Mater. Manuf. Process.

[15] Lin C.Y., Wang L.A., Chern G.W. (2001). Corrugated long-period fiber gratings as strain, torsion, and bending sensors. J. Lightwave Technol..

